# Belowground legacies of *Pinus contorta* invasion and removal result in multiple mechanisms of invasional meltdown

**DOI:** 10.1093/aobpla/plu056

**Published:** 2013-12-19

**Authors:** Ian A. Dickie, Mark G. St John, Gregor W. Yeates, Chris W. Morse, Karen I. Bonner, Kate Orwin, Duane A. Peltzer

**Affiliations:** 1 Landcare Research, Lincoln, New Zealand; 2 Bio-Protection Research Centre, Lincoln University, Lincoln, New Zealand; 3 Agriculture and Agri-Food Canada, Ottawa, Canada

**Keywords:** Biogeochemical processes, biological invasions, ecosystem function, ectomycorrhizas, facilitation, fungal : bacterial ratio, legacy effects, plant–soil interactions, removal effects

## Abstract

Plant invasions can change soil biota and nutrients in ways that drive subsequent plant communities, particularly when co-invading with belowground mutualists such as ectomycorrhizal fungi. These effects can persist following removal of the invasive plant and, combined with effects of removal *per se*, influence subsequent plant communities and ecosystem functioning. We used field observations and a soil bioassay with multiple plant species to determine the belowground effects and post-removal legacy caused by invasion of the non-native tree *Pinus contorta* into a native plant community. *Pinus* facilitated ectomycorrhizal infection of the co-occurring invasive tree, *Pseudotsuga menziesii*, but not conspecific *Pinus* (which always had ectomycorrhizas) nor the native pioneer *Kunzea ericoides* (which never had ectomycorrhizas). *Pinus* also caused a major shift in soil nutrient cycling as indicated by increased bacterial dominance, NO_3_-N (17-fold increase) and available phosphorus (3.2-fold increase) in soils, which in turn promoted increased growth of graminoids. These results parallel field observations, where *Pinus* removal is associated with invasion by non-native grasses and herbs, and suggest that legacies of *Pinus* on soil nutrient cycling thus indirectly promote invasion of other non-native plant species. Our findings demonstrate that multi-trophic belowground legacies are an important but hitherto largely unconsidered factor in plant community reassembly following invasive plant removal.

## Abstract (precis)

Invasive plants alter plant communities and transform landscapes aboveground, but also have strong belowground effects that are potentially even more important to ecosystem outcomes. Using management treatments of the widespread invasive tree, Lodgepole Pine, we find that pines and pine removal transform belowground ecosystems, increasing ectomycorrhizal inoculum and driving a change from slow-cycling fungal-dominated soils to fast-cycling bacterial-dominated soils with increased nutrient availability. This results in increased growth of graminoids, particularly exotic grasses, and facilitation of Douglas-fir establishment, hindering ecosystem restoration. The results highlight the importance of considering multiple species interactions in invasion, particularly in terms of belowground legacies.

## Introduction

Non-native invasive plants can alter plant communities and transform landscapes in obvious and profound ways (Pejchar and Mooney 2009; Rundel *et al*. 2014). Visual effects of invasive plants on landscapes can often be rapidly reversed by removal of the invader. However, invasive plants also have major belowground effects by changing soil biota and altering biogeochemical processes (Ehrenfeld 2010). These belowground effects may be more difficult to reverse, resulting in both biotic and abiotic legacies that can drive changes in subsequent plant community assembly, structure and ecosystem processes (Suding *et al*. 2013). Management of invasive plants may further augment some of these legacies, at least in the short term, for example through large pulses of organic matter inputs or disruption of plant–soil feedbacks (e.g. Symstad 2004; Martin *et al*. 2010). Although the belowground legacies of invasive nitrogen (N)-fixing plants are well documented (e.g. Vitousek *et al*. 1987; Liao *et al*. 2008), the importance of legacies involving other major soil symbioses is poorly understood (Nuñez and Dickie 2014).

Invasive pines (Pinaceae) are one of the most widespread invasive trees globally (Richardson and Rejmánek 2004; Procheş *et al*. 2012), and several lines of evidence suggest that they may have strong belowground legacies (Chapela *et al*. 2001; Reich *et al*. 2005; Nuñez and Dickie 2014). The Pinaceae have recalcitrant, low-calcium litter compared with other tree species, resulting in a reduction of soil pH and accumulation of organic matter in surface horizons (Reich *et al*. 2005). Because the Pinaceae are ectomycorrhizal, the establishment of pines is frequently associated with large changes in both soil biota and nutrient cycling (Nuñez and Dickie 2014). The establishment of ectomycorrhizal trees in plantations is associated with increased mineralization of organic P and N and a loss of soil carbon (C) in deeper soil layers (Chapela *et al*. 2001). Although less well studied than plantations, similar processes can also occur in self-established ectomycorrhizal trees (Dickie *et al*. 2011), suggesting that these effects are driven by the presence of the tree rather than the activity of planting or associated plantation management. In particular, Dickie *et al*. (2011) demonstrated that *Pinus nigra* invasion into grassland was associated with a loss of soil carbon (C) in mineral soil, increased P availability and rapid declines in soil invertebrate diversity.

Dickie *et al*. (2011) also observed increases in bacterial dominance of soil food webs following *P. nigra* invasion. This last result is at odds with the more general prediction that forest ecosystems should have greater fungal dominance of soil energy channels than grasslands (Imberger and Chiu 2001; Harris 2009) and observations of higher fungal dominance in young planted *Pinus radiata* compared with pasture (Macdonald *et al*. 2009). This discrepancy between studies remains unresolved, but is likely to be important for understanding ecosystem function. Bacterial dominated food webs are top-down controlled with increased bacterial dominance of energy channels regulated by predators such as nematodes (Wardle 2002), whereas the fungal-dominated energy channel is bottom-up controlled (i.e. resource limited). If pines increase fungal dominance, soil nutrient cycling is likely to slow and nutrients would be held in fungal biomass and become less available to plants. Conversely, if pines result in increased dominance of the bacterial channel, this is likely to increase bacterial-feeding nematodes and other predators, rather than bacteria *per se*, and thus result in faster nutrient cycling and increased nutrient availability (Wardle 2002).

Many of the belowground effects of pines are driven by ectomycorrhizal fungal symbionts co-introduced and co-invading with pines (Schwartz *et al*. 2006; Pringle *et al*. 2009). Invasive Pinaceae and their ectomycorrhizal mutualists frequently establish into grasslands and shrublands dominated by arbuscular mycorrhizal associations; as a consequence, the novel enzymatic capabilities of ectomycorrhizal fungi may cause fundamental shifts in soil nutrient cycling, C pools and mineralization of organic nutrients (Chapela *et al*. 2001; Chen *et al*. 2008; Orwin *et al*. 2011). Particularly over the initial period of *Pinus* establishment, it is possible that this mineralization of organic nutrients combined with high levels of C input may result in increased N and P availability to other plants, but this possibility has not been investigated.

Once established, it is likely that the presence of ectomycorrhizal fungi around established *Pinus* may facilitate further *Pinus* invasion, thus creating a positive feedback loop (Thiet and Boerner 2007). Facilitation of seedling mycorrhizal formation by established plants is commonly observed at forest margins (Dickie *et al*. 2005, 2012) and in early primary (Nara and Hogetsu 2004) and secondary succession (Thiet and Boerner 2007). A similar pattern is observed at the edge of *Pinus* plantations, with increased ectomycorrhizal infection of seedlings near plantation margins (Nuñez *et al*. 2009). Facilitation of ectomycorrhizal infection can also occur among plant species, where a pioneering species facilitates ectomycorrhizal infection of later establishing species (Horton *et al*. 1999), potentially increasing the competitive dominance of ectomycorrhizal plants with shared symbionts.

Many studies demonstrating plant effects on soil biota or properties have suggested that plant-soil feedbacks are important in driving above and belowground processes, yet few studies have completed the loop by demonstrating the aboveground consequences of these belowground changes (Suding *et al*. 2013). Here we determine how the effects of an invasive tree on soil biota, soil chemistry and ectomycorrhizal fungal inoculum combine to influence subsequent plant communities. Management of invasive trees typically involves removal of trees, but with little or no consideration given to how belowground legacies influence the regeneration of native vegetation, with the notable exception of N-fixing trees (e.g. Heneghan *et al*. 2006). More generally, complex multi-trophic processes in invasion, which typify belowground legacies, have been poorly recognized (Simberloff and Von Holle 1999; Nuñez *et al*. 2013). To address these issues, we studied the widespread non-native tree species *Pinus contorta* in New Zealand using a site where different management histories have resulted in *Pinus* invasion being prevented, reduced, allowed or allowed and then subsequently removed*.* These treatments, in combination with a bioassay of plant–soil interactions, enabled us to test three interlinked hypotheses:

- Invasive *P. contorta* facilitates the ectomycorrhizal infection of ectomycorrhizal plants, including conspecifics, the con-familial invasive *Pseudotsuga menziesii* and the native ectomycorrhizal pioneer *Kunzea ericoides*, resulting in increased dominance, nutrient uptake and growth of ectomycorrhizal plant species relative to arbuscular mycorrhizal and non-mycorrhizal species.
- Management history that permits the formation of closed-canopy *P. contorta* results in greatly increased soil C : N ratios, increased fungal dominance of soil biota, greater immobilization of N and reduced N uptake of all plants. Furthermore, the effects of these soil changes will have greater negative effects on arbuscular mycorrhizal and non-mycorrhizal species compared with ectomycorrhizal species, due to a lower ability of arbuscular mycorrhizal plants to utilize organic nutrients (Read *et al.* 1989; Read 1991).
- *Pinus* invasion results in a loss of total, but an increase in available, P in soils; this should result in increased P uptake by all species (Chen *et al*. 2008).

We note that the expectation of increased fungal : bacterial dominance in Hypothesis 2 is based on the commonly reported increased fungal dominance under forest compared with grasslands (Imberger and Chiu 2001; Harris 2009; Macdonald *et al*. 2009), but is contrary to the results for *P. nigra* invasion into grasslands in New Zealand (Dickie *et al*. 2011). One goal of the present study was to determine if the findings of Dickie *et al*. (2011) could be repeated in a novel site with a different *Pinus* species.

## Methods

We studied an invasive population of *P. contorta* Loudon (lodgepole pine) at Craigieburn Forest Park, Canterbury, New Zealand (43°9′04″S, 171°43′52″E, ∼800 m elevation, 1447 mm mean annual rainfall with >100 mm in most months, mean summer maximum 32.9 °C, mean winter minimum −8.6 °C; soils 10–15 cm depth yellow-brown earths over greywacke/argillite rock). The study system was dominated by the ectomycorrhizal tree *Fuscospora cliffortioides* (Hook.f.) Heenan & Smissen (formerly *Nothofagus solandri* var. *cliffortioides*) prior to burning by Māoris in the 13th century and European settlers in the 19th century, and the subsequent introduction of grazing animals (primarily *Ovis aries*). This produced relatively large areas of native tussock grassland and shrubland, which currently comprise about a quarter of New Zealand's land area. A large number of non-native tree species were planted from the 1950s to the early 1980s for forestry trials, re-vegetation and erosion control plantings, of which *P. contorta* and *P. menziesii* have become particularly invasive (Ledgard and Baker 1988; Ledgard 2001).

**Figure 1.**
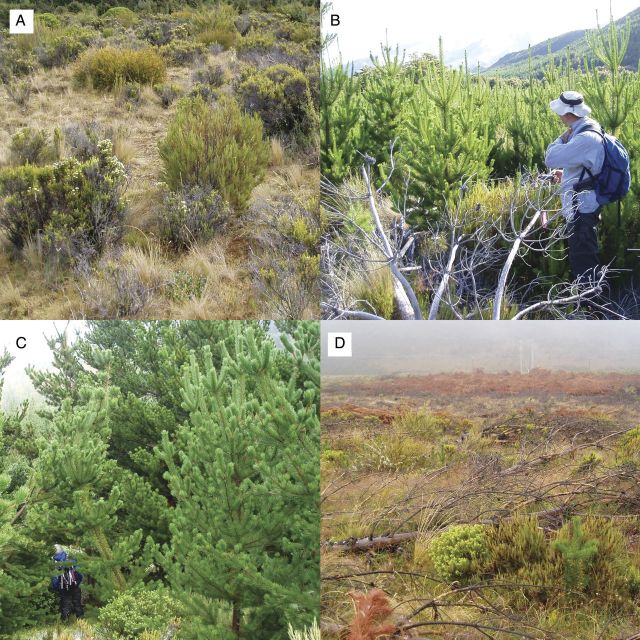
Examples of the four levels of invasive *P. contorta* management (A) Seedling-Removal, with dominance by native shrubs, herbs and grasses along with some invasive herbs, (B) Sapling-Removal, where the periodic removal of *P. contorta* has prevented closed-canopy formation (this photo taken 2 years after sampling was conducted), (C) No-Removal, with tall closed-canopy *Pinus* and (D) Tree-Removal, where closed-canopy *Pinus* was cut 3 years prior to this study.

We established 24 sampling plots spanning the gradient of *Pinus* abundance created by an existing spatial mosaic of management activity of *P. contorta* establishing into native-dominated grassland/shrubland (Fig. 1)*.* Candidate study plots (20 × 20 m) were established every 40 m along transects running perpendicular to the slope across the area, covering a total area of ∼1 km × 300 m. The cover of *P. contorta* within each plot was estimated visually, and a stratified random sample of 24 plots was retained from a total pool of 45 candidate plots. This design achieved an efficient spread of sampling effort across the range of *P. contorta* cover present. Six plots fell into areas where continuous management had prevented *Pinus* invasion as evidenced by the presence of infrequent and small *Pinus* stumps (<5 cm basal diameter) and pulled seedlings (<2 cm basal diameter). Hereafter, these are referred to as ‘Seedling-Removal’; we consider them to be the closest approximation to an un-invaded ecosystem. Percent cover of *P. contorta* was <1 %. Seven plots fell into areas where periodic control efforts had resulted in *Pinus* growing to *ca*. 10 cm basal diameter and 3–5 m height before removal with subsequent reinvasion resulting in pines 1–2.5 m in height, hereafter termed ‘Sapling-Removal’, with a mean 20.2 % cover of *P. contorta*. Six plots were in areas where no control efforts had taken place, resulting in closed-canopy *Pinus* stands of *ca*. 10 m height and 147.6 % cover (summed across height tiers; ‘No-Removal’), and five plots fell into areas where a similar closed-canopy *Pinus* forest had been removed *ca*. 3 years prior to our measurement (‘Tree-Removal’), with 2.3 % *P. contorta* cover. All control operations had been performed by hand felling with chainsaws, with no removal of biomass and minimal disturbance to soils. We believe that the plots have been subject to invasion by *P. contorta* for approximately the same total time, with anecdotal evidence of establishment since at least 1975 (N. Ledgard, Scion NZ, pers. comm.; available at: https://www.youtube.com/watch?v=AEm9l3Yffgo; accessed 5 August 2014), and extensive invasion by 1988 (Ledgard and Baker 1988).

### Plant communities

All vascular plant species rooted within plots were identified to species, and percentage cover was measured using cover classes following standard vegetation survey protocols (Hurst and Allen 2007). Cover was measured within each of four height tiers (0–0.3, 0.3–2, 2–5, 5–12 m), such that total cover of a species can sum to >100 %.

### Soils

Soils for chemical and biological analysis were homogenized from five locations within each plot (at the centre and at four orthogonal points 7.07 m from the centre) using a custom-made 65-mm diameter metal coring device to sample the top 100 mm of mineral soil. This depth is the most relevant during early establishment of plant species. Litter was generally sparse, except under the densest pine in the No-Removal treatment, and was not included in samples. Even under closed-canopy pine, there was an abrupt transition between litter and mineral soil and no F or H layer had developed. Soil samples were analysed for pH, total C, total N, KCl extractable NH_4_–N and NO_3_-N, total P and Bray-extractable P.

Microbial communities were characterized by phospholipid fatty acid (PLFA) profiling, using gas chromatography. Phospholipid fatty acid profiling was chosen over DNA-based methods as it is uniquely able to directly compare bacterial and fungal biomass (Baldrian *et al*. 2013). We used 18 : 2ω9, 12 to represent fungi, 16 : 1ω5 to represent arbuscular mycorrhizal fungi and summed cy-17 : 0, cy-19 : 0, i-15 : 0, a-15 : 0, i-16 : 0, i-17 : 0 and 16 : 1ω7 to represent bacteria. Soil invertebrates were extracted and enumerated using the tray method (Yeates and Bongers 1999) from *ca*. 80 g (dry mass) of each soil sample. All enchytraeids and nematodes within the sample were counted. Approximately 100 individual nematodes from each sample were identified to nominal genus and assigned to functional guilds based on feeding types. For both PLFA and nematode data we calculated fungal : bacterial dominance as *F*/(*F*+ *B*) where *F* is the fungal PLFA marker or fungal-feeding nematode abundance and *B* is the sum of bacterial PLFA markers or bacterial-feeding nematode abundance. This has the advantage of a symmetric distribution under either fungal or bacterial dominance and is equivalent to the commonly calculated nematode-channel ratio for fungal dominance of nematode-feeding groups, but differs from a straight *F*: *B* ratio often reported in PLFA data as *F*/*B*.

### Plant growth responses: *ex situ* bioassay

To measure the response of different plant species to treatment effects, we planted six plant species that co-occur in the field and represent a range of mycorrhizal status and growth forms into intact soil cores (Table 1). From the centre of each of the 24 plots we removed six, intact, 110-mm depth soil cores using a 65-mm diameter metal-coring device directly into 65-mm PVC tubes on 11 December 2007, at the start of the Austral summer. The top 10 mm of soil was sliced from the top of each sample with a knife to remove plants and root crowns. Sampling equipment was sterilized with a 10 % household bleach solution between plots. Soil cores were immediately wrapped in a aluminium foil, stored in a cool storage box and returned within 8 h to a 4 °C refrigerator. The subsequent day, cores were moved to an unheated greenhouse, aluminium foil was removed, bottoms of tubes were covered with 1.5-mm mesh screen secured by adhesive tape and cores were placed into individual 100 × 100 mm aluminium trays to prevent any water flow between pots (which might have transmitted soil biota).

**Table 1.** Species utilized in bioassay. Here we use ‘non-mycotrophic’ to refer to a plant species that is *typically* a non-host of mycorrhizal fungi, as opposed to ‘non-mycorrhizal’, which we use for individual plants that do not have any mycorrhizal infection present but that typically do host mycorrhizal fungi.

	Origin	Mycorrhizal status	Growth form
*P. contorta*	Exotic	Ectomycorrhizal	Tree
*P. menziesii*	Exotic	Ectomycorrhizal	Tree
*K. ericoides*	Native	Dual ecto/arbus	Tree
*Coprosma robusta*	Native	Arbuscular	Shrub
*Carex comans*	Native	Non-mycotrophic	Sedge
*Poa cita*	Native	Arbuscular	Grass

Soil cores were watered to field capacity and each was planted with two seedlings of one species (Table 1). Seeds were germinated in sterile perlite before being transplanted 1–4 weeks following germination (sowing dates were staggered to synchronize planting on 12 December 2007). On 5 February 2008, one randomly selected seedling was removed in the case of both seedlings surviving. Seedlings were harvested on 15 May 2008. Roots were washed from the soil, and both roots and shoots were dried and weighed. All aboveground shoot tissue was ground and analysed for N and P content. For the three ectomycorrhizal species (*Pinus, Pseudotsuga* and *Kunzea*), ectomycorrhizal infection was quantified under a stereomicroscope with the presence of mantle confirmed under compound microscopy. All terminal roots (‘root tips’) on seedlings were evaluated as being either non-mycorrhizal or mycorrhizal. Arbuscular mycorrhizal infection was not quantified.

### Statistics

Field data were analysed by analysis of variance and Tukey's HSD tests to determine significance, with arcsine square-root transformation of ratio data. Plant community compositional shifts among management strategy treatments were determined by summarizing species compositional data as ordination axes using non-metric multi-dimensional scaling (NMDS). We tested the significance of management treatment using nonparametric permutational multivariate analysis of variance (PERMANOVA) as implemented in the function adonis, vegan package, R version 3.01 (R Core Team 2013). Ectomycorrhizal infection of seedlings was tested for the presence/absence of ectomycorrhizas using a *χ*^2^ test. *Pinus* and *Pseudotsuga* responses to ectomycorrhizal infection were tested using ANCOVA with terms for treatment, mycorrhizal infection (% of root tips) and their interaction with non-significant terms sequentially removed. Plant growth responses were analysed by comparing the three treatments with pine establishment (Sapling-Removal, No-Removal, Tree-Removal) with the Seedling-Removal treatment. Biomass (log transformed) and N- and P-concentration responses were calculated as the difference between the treatment-mean value and the mean value from the Seedling-Removal treatment divided by the maximum of the treatment or Seedling-Removal values. This puts all seedling species on a similar response scale for the effect of pine establishment on subsequent seedling growth and nutrient concentrations. It also provides a parallel metric to a common calculation of plant–soil feedbacks with symmetry around zero (e.g. Diez *et al*. 2010). We also tested results with log-response ratios (log (treatment/control)) and note where this makes a qualitative difference to the outcome (one instance).

## Results

### Vegetation survey

**Figure 2.**
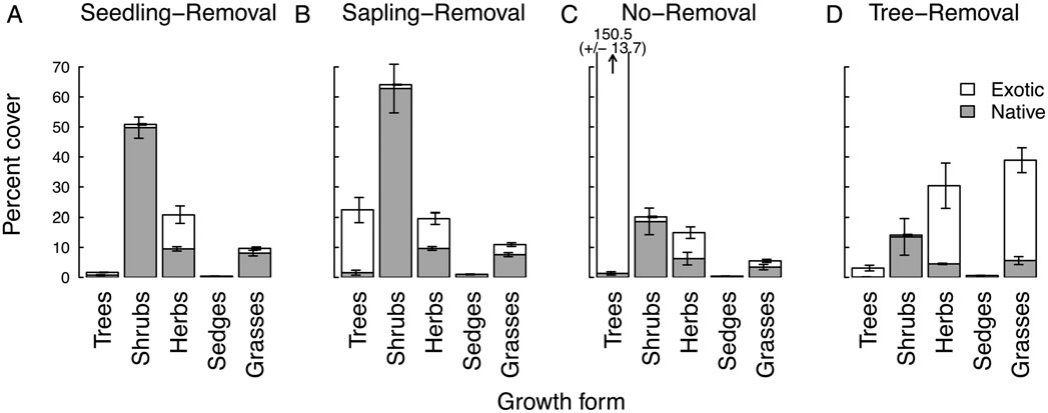
Vegetation structure as percent cover of native (grey bars) and non-native (open bars) plants of different growth forms. Cover values are summed across different height tiers, such that total cover can exceed 100 %. Error bars indicate standard errors within native (offset to left) and exotic (offset to right) categories.

Vegetation was dominated by native shrubs with a smaller component of herbs (slightly >50 % exotic) and native grasses where invasive *P. contorta* was prevented from establishing (Seedling-Removal; Fig. 2A). The rank order of the dominance of different plant groups was similar under Sapling-Removal and No-Removal, but with a substantial reduction in shrub cover and increase in tree cover in the No-Removal treatment (Fig. 2B and C). Vegetation structure in the Tree-Removal treatment, in which *Pinus* had established and then been removed, shifted strongly with increases in non-native grasses (*P*_3,20_ < 0.0001, Tree-Removal significantly different from all other treatments, no other contrasts significant) and non-native herbs (*P*_3,20_ = 0.019; Fig. 2D, Tree-Removal significantly different from Sapling-Removal and No-Removal, no other contrasts significant). There was also significantly lower cover of native shrubs under both No-Removal and Tree-Removal relative to Seedling-Removal and Sapling-Removal (*P*_3,20_ < 0.0001).

**Figure 3.**
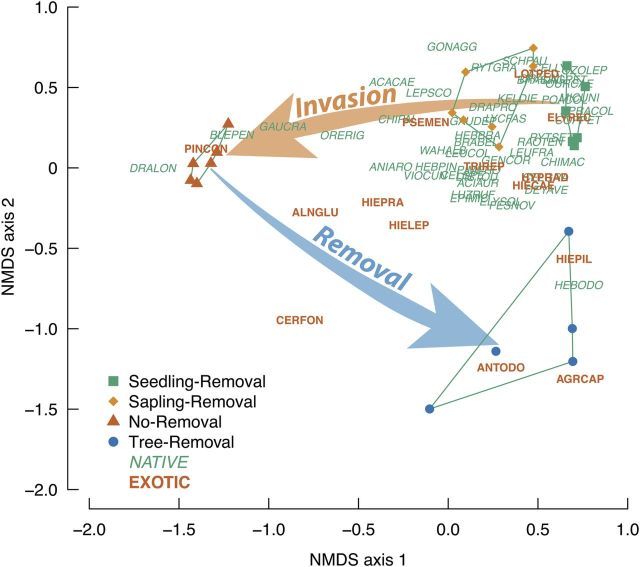
Non-metric multi-dimensional scaling ordination of plant communities in plots under the four management regimes (points) and the position of all native and non-native (exotic) plant species found in more than three plots (six letter codes as the first three letters of genus and species, full names are in **Supporting Information**). Lines show the outer hull around all plots within a treatment. Arrows indicate the progressive change with increased *P. contorta* invasion along Axis 1, and the return of community composition on Axis 1 but strongly shifted plant community along Axis 2 following *P. contorta* removal.

Within-plot plant species richness was 42.3 ± 0.67 (mean ± standard error) in Seedling-Removal and 50.6 ± 1.8 species in Sapling-Removal, but significantly lower in No-Removal (33.8 ± 4.0) and Tree-Removal (34.8 ± 2.2) treatments (*P*_3,20_ = 0.00022). Under the Seedling-Removal treatment, vegetation was dominated by the native Ericaceous shrubs *Dracophyllum uniflora* and *D. longifolium* (36 % cover), the non-native herb *Hieracium pilosella* (9 % cover), the native shrub *Ozothamnus letophyllus* (6 %) and the native grasses *Chionochloa* spp (4 %) and *Poa colensoi* (3 %). Non-metric multi-dimensional scaling analysis showed a strong movement along NMDS axis 1 with increasing *Pinus* invasion (Fig. 3). Under the Tree-Removal treatment, axis 1 scores returned to similar values to the Seedling-Removal treatment, but plots were strongly separated on axis 2, reflecting dominance of non-native grasses *Agrostis capillaris* (22 % cover), *Anthoxanthum odoratum* (12 %) and the non-native herb *H. pilosella* (19 %). Permutational multivariate analysis of variance showed a strong effect of treatment (*P*_3,20_ < 0.001, *R*^2^ = 0.76). This included a significant treatment difference between Seedling-Removal and Tree-Removal (*P*_1,9_ = 0.003, *R*^2^ = 0.54).

### Soil chemistry and biota

Soil pH was significantly lower under No-Removal than under Seedling-Removal or Sapling-Removal treatments, but soil C, total N and C : N did not differ among treatments (Table 2). Despite the lack of change in total N, there was a 14- to 17-fold increase in NO_3_-N in the Tree-Removal treatment compared with the other treatments (Table 2). Total P was 1.5× higher in the Tree-Removal treatment than in the Sapling-Removal treatment, but no other contrasts were significant (Table 2). Available P, in contrast, showed up to a 3.2× increase in Tree-Removal relative to Seedling-Removal or Sapling-Removal treatment and was also 2.5× higher in the No-Removal relative to Seedling-Removal treatment (Table 2).

**Table 2.** Soil abiotic and biotic responses to pine management strategy, means and standard errors. Significant responses shown in bold, values sharing the same superscript letter are not significantly different (*P* < 0.05, Tukey's HSD test). *P* value for NO_3_-N, total *P* and available P based on log-transformation to reduce inequality of variances. ^†^Both PLFA fungal dominance and nematode feeding fungal channel dominance calculated as *F*/(*F*+ *B*). A value of 1 would indicate complete fungal dominance, and a value of 0 complete bacterial dominance. Mean separation and *P* value are based on arcsine square-root transformation.

	Pine removal treatment				
	Seedling-Removal	Sapling-Removal	No-Removal	Tree-Removal	*P*_3,20_
Soil chemistry					
pH	**5.2**±**0.031^a^**	**5.1**±**0.024^a^**	**4.9**±**0.026^b^**	**5.0**±**0.12^ab^**	**0**.**0058**
C (%)	6.2 ± 0.22	5.8 ± 0.26	5.5 ± 0.34	6.0 ± 0.37	0.46
Total N (%)	0.35 ± 0.014	0.31 ± 0.017	0.30 ± 0.025	0.33 ± 0.029	0.34
C : N ratio	18 ± 0.35	19 ± 0.54	19 ± 0.66	18 ± 0.66	0.45
NH_4_–N (mg kg^−1^)	13 ± 3.1	10 ± 2.2	12 ± 1.0	19 ± 7.0	0.41
NO_3_-N (mg kg^−1^)	**0.34**±**0.03^a^**	**0.26**±**0.01^a^**	**0.41**±**0.13^a^**	**5.8**±**4.5^b^**	**0**.**0064**
Total P (mg kg^−1^)	**770**±**67^ab^**	**560**±**23^a^**	**720**±**120^ab^**	**840**±**59^b^**	**0**.**035**
Available P (mg kg^−1^)	**5.9**±**1.3^a^**	**6.1**±**0.92^ab^**	**15**±**4.2^bc^**	**19**±**4.4^c^**	**0**.**0018**
Soil microbial community (PLFA, relative C19)					
Total PLFA (nmol g^−1^)	268 ± 16	277 ± 7.2	292 ± 14	266 ± 19	0.55
Fungi (nmol g^−1^)	**44**±**4.0^a^**	**48**±**2.3^a^**	**44**±**4.0^a^**	**26**±**5.2^b^**	**0**.**0051**
Bacteria (nmol g^−1^)	76 ± 3.9	76 ± 2.3	88 ± 5.6	82 ± 2.8	0.11
Fungal dominance^†^	**0.36**±**0.016^a^**	**0.38**±**0.014^a^**	**0.33**±**0.027^a^**	**0.24**±**0.031^b^**	**0**.**0010**
AMF (nmol g^−1^)	11 ± 0.61	11 ± 0.70	10 ± 1.4	12 ± 1.3	0.44
Soil invertebrate community					
Enchytraeids (g^−1^)	**0.24**±**0.12^a^**	**0.36**±**0.07^a^**	**0.90**±**0.22^a^**	**2.4**±**0.79^b^**	**0**.**0012**
Nematodes (g^−1^)	4.1 ± 1.0	5.0 ± 1.3	5.3 ± 1.1	8.8 ± 2.1	0.15
Bacterial-feeding nematodes (g^−1^)	**1.3**±**0.34^a^**	**1.5**±**0.19^a^**	**3.3**±**0.71^ab^**	**4.7**±**1.6^b^**	**0**.**014**
Fungal-feeding nematodes (g^−1^)	0.87 ± 0.34	0.60 ± 0.12	0.15 ± 0.04	0.41 ± 0.25	0.14
Fungal channel dominance^†^	**0.36**±**0.05^a^**	**0.29**±**0.03^ab^**	**0.05**±**0.02^c^**	**0.15**±**0.11^bc^**	**0**.**0019**
Plant feeding + associated (g^−1^)	1.2 ± 0.3	2.2 ± 1.2	0.98 ± 0.16	1.6 ± 0.47	0.70
Omnivorous nematodes (g^−1^)	0.51 ± 0.04	0.49 ± 0.1	0.68 ± 0.28	1.5 ± 0.59	0.058
Predacious nematodes (g^−1^)	0.20 ± 0.043	0.24 ± 0.10	0.25 ± 0.06	0.50 ± 0.19	0.21

Total microbial PLFA did not change with treatment. There were, however, large shifts in microbial community composition in the Tree-Removal treatment relative to other treatments (Table 2), with a decline in the quantity of fungal PLFA markers, a marginally significant increase in bacterial PLFA markers and a shift towards increasing bacterial dominance.

Soil invertebrate communities showed a shift to increased bacterial-feeding dominance under No-Removal and Tree-Removal (Table 2). This was driven largely by a 2.5–3.6× increase in bacterial-feeding nematodes relative to Seedling-Removal. Enchytraeids increased 10× in abundance in the Tree-Removal treatment relative to the Seedling-Removal treatment and there was a marginally significant increase in omnivorous nematodes.

### Ectomycorrhizal infection of seedlings

In the *ex situ* bioassay of soils from all plots, ectomycorrhizas were found on 22 of the 24 *Pinus* seedlings, with the two exceptions being in the Seedling-Removal treatment (no significant treatment effect; *χ*^2^ test, *P* = 0.15). The percentage of root tips infected (29.6 ± 4.4 %) in *Pinus* with ectomycorrhizas showed no significant difference among treatments.

No *Pseudotsuga* seedlings were ectomycorrhizal in soils from the Seedling-Removal or Sapling-Removal treatments (*n* = 6 for both), three out of six *Pseudotsuga* were ectomycorrhizal in the No-Removal treatment and two out of five *Pseudotsuga* were ectomycorrhizal in the Tree-Removal treatment (marginally significant difference in the *χ*^2^ test for differences across all four treatments, *P* = 0.057). The marginal significance may have reflected low sample size. When the two treatments where pines were actively controlled (i.e. Continuous and Sapling-Removal; 0 out of 12 were ectomycorrhizal) and the two treatments where pines had formed closed-canopy forest (i.e. No-Removal and Tree-Removal; 5 out of 11 were ectomycorrhizal) are contrasted, the difference is highly significant (*χ*^2^ test, *P* = 0.0055). When ectomycorrhizal, *Pseudotsuga* seedlings had 50.6 ± 15.2 % of root tips infected, with no significant treatment difference between the No-Removal and Tree-Removal treatments. Ectomycorrhizas were not found on any *Kunzea* seedlings in the experiment.

Seedling biomass was positively correlated with ectomycorrhizal infection for both *Pinus* (log biomass, *P*_1,22_ = 0.036, *R*^2^_adj_ = 0.15, no significant treatment effect or interaction) and *Pseudotsuga* (linear, *P*_1,21_ = 0.006, *R*^2^_adj_ = 0.48, no significant treatment effect or interaction). Phosphorus concentrations of both seedlings were also positively correlated with ectomycorrhizal infection for both *Pinus* (*P*_1,20_ = 0.019, *R*^2^_adj_ = 0.21, no significant treatment effect or interaction) and *Pseudotsuga* (*P*_1,21_ = 0.011, *R*^2^_adj_ = 0.23, no significant treatment effect or interaction). Nitrogen was positively correlated with ectomycorrhizal infection for *Pinus* (*P*_1,14_ = 0.015) with a significant effect of tree-removal treatment (*P*_3,14_ = 0.0067), discussed below, and interaction term (*P*_3,14_ = 0.044; model *R*^2^_adj_ = 0.59). The significant interaction was driven by a more positive effect of ectomycorrhizal infection in the No-Removal compared with other treatments. Nitrogen was not correlated with ectomycorrhizal infection for *Pseudotsuga* (*P*_1,18_ = 0.72).

### Seedling biomass and nutrient responses

**Figure 4.**
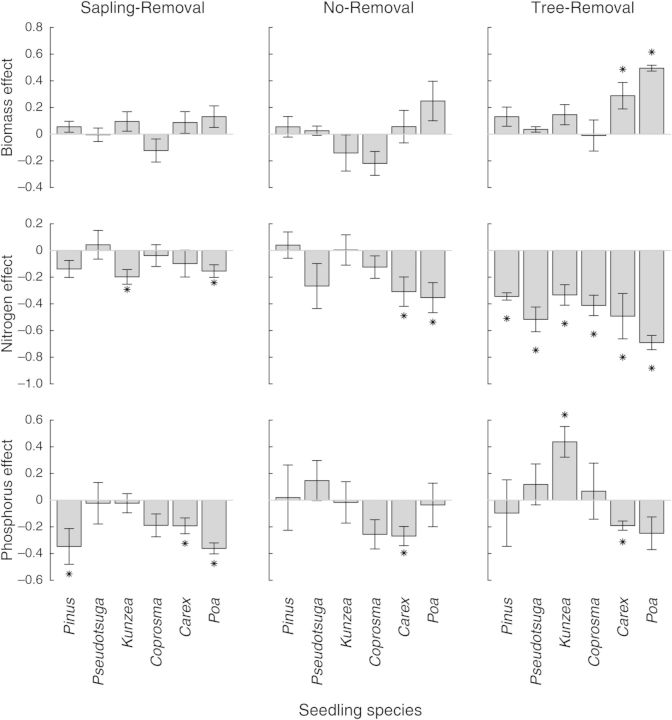
Biomass, percent N and percent P change in bioassay seedlings of six species planted in soils from Sapling-Removal, No-Removal and Tree-Removal relative to Seedling-Removal. Scale bars show mean and standard error of (treatment—Seedling-Removal)/maximum (treatment, Seedling-Removal). Values significantly different from zero are indicated by asterisks. The significant increase in *Kunzea* P in the Tree-Removal treatment was only marginally significant as a log-response ratio (log (treatment/control)). All other results were qualitatively similar.

The only significant effects of soil origin on seedling biomass in the *ex situ* bioassay were for *Carex* and *Poa*, both of which had increased biomass in the Tree-Removal compared with the Seedling-Removal soils (Fig. 4).

**Figure 5.**
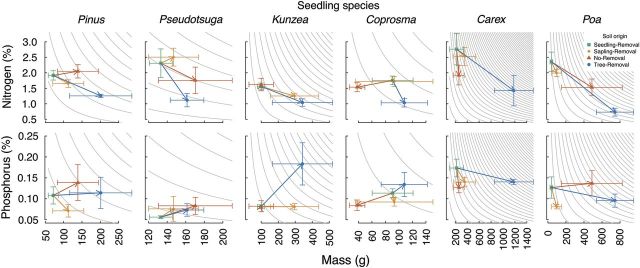
Vector analysis of foliar percent N and P as a function of seedling mass of each species in the *ex situ* bioassay showing means and standard errors for each treatment. Grey isoclines indicate constant nutrient content, and arrows indicate change relative to the Seedling-Removal treatment. Declines in nutrient concentration occur despite increased total nutrient content due to the disproportionate increase in plant mass.

All six species had significantly reduced N concentrations in the Tree-Removal treatment compared with Seedling-Removal. *Poa* and *Carex* also had lower N concentrations in the No-Removal treatment, and *Poa* and *Kunzea* had lower N concentrations in the Sapling-Removal treatment relative to the Seedling-Removal. Vector analysis diagrams (Fig. 5) suggest that the lower N concentrations are due to biomass increases being substantially greater than total N increases, resulting in decreased concentrations of N despite increasing total N.

Phosphorous concentrations of seedlings were reduced in *Pinus, Carex* and *Poa* in Sapling-Removal, and also in *Carex* in No-Removal and Tree-Removal soils relative to Seedling-Removal. Only *Kunzea* in the Tree-Removal treatment had significantly increased P concentration. Vector analysis diagrams (Fig. 5) showed that the significant decreases in P shown in Fig. 4 were mostly due to increased biomass rather than decreased total P.

## Discussion

Plant–soil feedbacks are widely studied, yet relatively few studies have demonstrated both the mechanistic basis of these feedbacks and their consequences for aboveground communities (Bever *et al*. 2010; Suding *et al*. 2013). Our study of tree invasions into native grassland demonstrates two important plant–soil feedback mechanisms at work: increased ectomycorrhizal inoculum and altered soil biogeochemical cycling. Altered soil properties subsequently caused species-specific legacies for plant communities, resulting in a major shift in dominance among different plant growth forms. The alteration of soil biogeochemical cycling is particularly interesting because most prior studies have focussed on the effects of invasive N-fixing plants in N-limited systems (e.g. Vitousek *et al*. 1987; Heneghan *et al*. 2006; Liao *et al*. 2008; Nuñez and Dickie 2014), whereas *Pinus* is not N fixing. As one of the most invasive trees globally (Richardson and Rejmánek 2004; Procheş *et al*. 2012), understanding *Pinus* effects is inherently important, but we suggest that our results can also be generalized to other ectomycorrhizal invasive trees (Nuñez and Dickie 2014).

We expected that the establishment of invasive *Pinus* would cause major shifts in soil nutrient cycling, with increased P availability but also increased fungal dominance and N limitation relative to the predominantly native plant community present when *Pinus* was excluded (i.e. Seedling-Removal treatment). Invasive *Pinus* increased P availability, consistent with our expectation, but there were also unexpected large increases in labile N (NO_3_-N) following pine removal. Although based on a single point-in-time measurement, the increase in available P and NO_3_-N was reflected in increased seedling total N and total P (Fig. 5) and hence indicates an increase in total flux, not just pool size. Soil microbial trophic channels did not shift towards increased fungal dominance, but rather to increased dominance by bacteria, bacterial-feeding nematodes and enchytraeids. Unlike N-fixing invaders, invasive *Pinus* did not substantially add resources. Rather, *Pinus* appears to have switched soil function from a slow-cycling, more nutrient-conservative system to a fast-cycling, potentially more ‘leaky’ system (Hobbie 1992), with the same quantity of nutrients cycling more rapidly. This fundamental biogeochemical shift also contributes to the strong belowground legacies generated during invasion by *Pinus*, with subsequent effects on performance of co-occurring plant species. In particular, we observed large increases in the growth of graminoids (an arbuscular mycorrhizal grass and a non-mycotrophic sedge *Carex*) in soils from which *Pinus* had been removed. This belowground legacy observed in the greenhouse is consistent with field observations of increased dominance by non-native grasses and non-native herbaceous plants, species that are likely adapted to high-nutrient turnover, in plots where *Pinus* had been removed. More generally, these findings support the view that biogeochemical disruptions can facilitate plant invasions (e.g. Davis *et al.* 2000).

We also found ectomycorrhizal facilitation by invasive *Pinus,* but this effect differed among plant species. The establishment of closed-canopy *P. contorta* forest, whether intact (No-Removal) or removed (Tree-Removal), facilitated ectomycorrhizal infection of the con-familial invasive tree species *P. menziesii,* but not conspecific *Pinus* or the native shrub *K. ericoides*. This suggests a selective biotic feedback, whereby early plant invaders differentially facilitate the establishment or subsequent performance of later plant species through their effects on soil populations of symbionts. Shared symbionts may be particularly the case for con-familial trees that originate from the same geographical region, such as *P. contorta* and *P. menziesii*, which are known to naturally share fungal associates (Molina and Trappe 1992). *Pseudotsuga* was not a major component of current plant communities at the site (comprising <1 % cover) but is of particular conservation concern as the most shade tolerant of invasive conifers (Froude 2011).

### Effects of *Pinus* on soils and soil communities

The common expectation is that forest ecosystems, and particularly ectomycorrhizal forests, will have fungal-dominated, slow nutrient cycling soils (Imberger and Chiu 2001; Harris 2009). Our observation that invasive *Pinus* increased soil nutrient availability and the importance of the bacterial energy channel run counter to this expectation, although it is consistent with earlier observations of ectomycorrhizal *P. nigra* and *K. ericoides* successions (Dickie *et al*. 2011). The concept of forests as fungal dominated and grasslands as bacterial dominated is based primarily on comparisons of more mature forests with post-agricultural grasslands, but our results suggest that this may not apply more generally to early stages of woody succession or invasion into grass and shrub-dominated communities. Our results also suggest that *Pinus* invasion has increased energy flows to soil and a shift towards bacterial dominance, with concomitant increases in nutrient cycling rates (Hobbie 1992).

Increased bacterial dominance was expressed most strongly in increased bacterial predator densities rather than bacterial PLFA levels, reflecting the top-down regulation of the bacterial energy channel (Wardle 2002). Shifts towards dominance of the bacterial energy channel in soil food webs have been frequently associated with increased ecosystem productivity, e.g. in the presence of N-fixing plants (St John *et al*. 2012; de Vries *et al*. 2013). An increase in ecosystem productivity also commonly occurs in plant invasions, particularly invasions by woody plants (Liao *et al*. 2008). More rapidly cycling systems usually result in higher soil nutrient availability, consistent with our observation of large increases in NO_3_-N and available P. The dominance of bacterial-feeding nematodes and enchytraeids was particularly high in the Tree-Removal treatment. This may reflect a response to the large pulse of C and nutrients released after felling of pines 3 years prior to this study. Nonetheless, not all of the observed effects can be attributed to a post-removal nutrient flush as the nematode fungal channel dominance reached its nadir under intact pine (No-Removal). There was also no observed increase in soil C in the Tree-Removal treatment relative to other treatments, suggesting that any possible flush of organic C following removal was no longer present in the soil after 3 years. Rapid mineralization of C is consistent with the observed bacterial trophic dominance.

Increased availability of P has been observed in both planted and invasive *Pinus* (Chen *et al*. 2008; Dickie *et al*. 2011), as has a release of soil C (Chapela *et al*. 2001; Dickie *et al*. 2011). These changes may reflect the unique enzymatic capabilities of some ectomycorrhizal fungi, particularly the ability to acquire N and P from organic sources while obtaining C from their plant symbionts (Dickie *et al*. 2014). However, the dominance of native shrubs associated with ericoid mycorrhizal fungi in this system prior to invasion suggests that the ability to utilize organic nutrient sources is not a novel ecosystem function (Read and Perez-Moreno 2003). Rather, the relatively high growth rates of invasive *Pinus* may be associated with greater C allocation belowground, which may increase the rate of organic nutrient mineralization by microbes, but more detailed investigation is required. Our results, in combination, demonstrate that invasion by *Pinus* causes biogeochemical disruption through changes in macronutrients, as well as shifts in the soil biota controlling biogeochemical processes. The long-term persistence of changes in soil nutrient cycling remains uncertain, particularly given that pines increased the rate of nutrient cycling but did not increase nutrient stocks. Indeed, the initial state of the ecosystem is somewhat perplexing in having quite high levels of total N and P (C : N : P ratio 83 : 4.6 : 1) and only moderately acidic soils (pH 5.2), yet being dominated by relatively slow-growing Ericaceous shrubs. This may suggest that once the system has been disrupted into a high-nutrient cycling state, it may not quickly recover.

### Ectomycorrhizal facilitation

The establishment of closed-canopy invasive *Pinus* facilitated the ectomycorrhizal infection of *Pseudotsuga*, but not of *Pinus* or *Kunzea*. Despite having a broad receptivity to different species of ectomycorrhizal fungal symbionts, *Pseudotsuga* seedlings can be relatively slow to establish ectomycorrhizas in early succession (Borchers and Perry 1990; Davis *et al*. 1996; Horton *et al*. 1999; Ledgard 2004; Kazantseva *et al*. 2009). In its native range, *Pseudotsuga* appears to benefit from prior establishment of pioneering ectomycorrhizal plants (Borchers and Perry 1990; Horton *et al*. 1999). Our observation of increased mycorrhizal infection of *Pseudotsuga* in soils where closed-canopy *Pinus* had been present suggests that as an invasion process, the prior co-invasion of *Pinus* and its mycorrhizal fungi (Dickie *et al*. 2010) can result in greater ecosystem vulnerability to *Pseudotsuga* invasion. This is an important and potentially widespread example of sequential invasional meltdown, whereby one invasive species greatly increases the subsequent success or performance of other invaders, in this case through biotic interactions with mutualists (Simberloff and Von Holle 1999; Nuñez *et al*. 2013).

We did not observe strong effects of invasive *Pinus* on the establishment of ectomycorrhizal mutualists on either *Pinus* or *Kunzea* seedlings, with most *Pinus* and no *Kunzea* seedlings having ectomycorrhizas. The differences in mycorrhizal infection between *Pinus*, *Pseudotsuga* and *Kunzea* may reflect fungal host specificity. Inoculum of the ectomycorrhizal fungus *Rhizopogon* is likely widespread at this site from adjacent established *Pinus* plantations and is unusual among ectomycorrhizal fungi in having spore longevity of at least several years in soils (Bruns *et al*. 2009). The presence of a widespread spore bank may explain the widespread infection of *Pinus*, but as *Rhizopogon* has very high levels of host specificity (Molina and Trappe 1994) it may only benefit *Pinus. Kunzea*, in contrast, was never ectomycorrhizal in our greenhouse bioassay despite its known capability to form ectomycorrhizas (Orlovich and Cairney 2004). This may reflect an incompatibility with ectomycorrhizal fungi co-invading with pine. On the other hand, *Kunzea* has the ability to form arbuscular mycorrhizas as well as ectomycorrhizas (Moyersoen and Beever 2004), and it may be that greenhouse conditions favoured this alternative mycorrhizal type as other greenhouse studies have also failed to establish ectomycorrhizas on *Kunzea* (Davis *et al*. 2013).

### Belowground legacy effects on plant growth

The changes in plant community composition observed following the removal of *Pinus* represent a major functional shift from shrub-dominance to dominance by exotic herbs and grasses. Grasses were present in the Seedling-Removal treatment, but even within this broad functional category, *A. capillaris* is distinct as a rhizomatous grass while the dominant native grasses of the area are tussock forming. Once established, rhizomatous grasses have the potential to form dense swards that are more resistant to subsequent establishment of woody plants than tussock-forming grasses (D'Antonio and Vitousek 1992). *Hieracium pilosella*, the main invasive herb, also forms dense mats. A study of the invasive con-generic *H. lepidulum* in adjacent ecosystems suggested that the addition of *Hieracium* to the established native vegetation did not result in a loss of plant diversity (Meffin *et al*. 2010), but other studies have suggested a strong ability of *H. pilosella* to suppress native plant growth, particularly under high fertility and high light conditions (Moen and Meurk 2001). In part these discrepancies may reflect priority effects: an established native community may be resilient to *Hieracium* impacts, but an established *Hieracium* mat may be resistant to native re-establishment following disturbance.

The observed rapid increase in the abundance of non-native grasses and herbs following *Pinus* removal in the field could have been driven by multiple factors, including seed availability and an ability to respond to disturbance. Nonetheless, the large increase in graminoid growth in the greenhouse bioassay suggests that belowground biogeochemical legacies of *Pinus* are an important contributing factor. Graminoids and herbs tend to have a much greater plasticity in response to increased nutrient availability compared with woody plants, and this may be particularly the case for invasive grasses and herbs (Funk 2008). The increased availability of N and P following *Pinus* invasion may therefore be particularly beneficial to these functional groups.

### Removal versus species effect

Some of the treatment effects we observed were associated with both the No-Removal and Tree-Removal treatments, while others were observed only following Tree-Removal. For example, available P and bacterial dominance of nematode-feeding ratios were significantly higher in both the No-Removal and Tree-Removal treatments compared with the Seedling-Removal treatment. This suggests that these effects can be attributed to effects of the shift in plant community from native dominance to *Pinus* rather than *Pinus* removal activity or residual biomass left after removal. In contrast, the increase in available N and bacterial dominance measured by PLFA were seen only in the removal treatment. Effects seen only in the removal treatment may represent non-species-specific removal effects (nutrient pulses, disturbance) rather than *Pinus*-specific species effects. The increased bacterial dominance between No-Removal and Tree-Removal, which is driven by a reduction in fungi, may reflect a loss of *Pinus*-associated ectomycorrhizal fungal biomass following tree removal.

Treatment effects on plant communities were complex. Increased graminoid biomass was only statistically significant in the Tree-Removal treatment. Nonetheless, *Poa* spp. in particular had an increase in biomass almost as large under the intact *Pinus* No-Removal treatment as under the Tree-Removal treatment (Figs 4 and 5). Our interpretation is that increased P availability, bacterial dominance of nematode trophic channels and graminoid growth are likely driven by direct effects of *Pinus*, but that these effects may be further augmented by a large influx of leaf litter, fine roots and woody biomass following tree removal. This would be consistent with the *Pinus* uptake of both total P and recalcitrant P sources, incorporation into foliage and recycling through litter fall (all presence effects; Chen *et al*. 2008; Dickie *et al*. 2011). Tree removal may further augment these effects by creating a sudden influx of litter and fine roots. In contrast, increased N availability and decreased fungal biomass as measured by PLFA appear likely to be a removal effect, as neither was observed in the other treatments. Soil pH appeared to be the only *Pinus* effect that was reversible within three years of removal. Taken as a whole, the results suggest that both direct effects of *Pinus* and removal effects are important belowground legacies. Where effects can be attributed specifically to removal (e.g. increased NO_3_-N), there may be potential to mitigate effects through different removal strategies.

## Conclusions

The regime shift driven by *Pinus* invasion has the potential to represent a novel stable state (Folke *et al*. 2004). Periodic removal of trees does not result in restoration of native plants, at least within 3 years, but rather dominance of non-native grasses and herbaceous plants, likely driven by increased nutrient availability. Even re-measuring the site in 2014, 9 years following *Pinus* removal, we found no native regeneration (data not shown). This community is, in turn, likely to be re-invaded by *Pinus*, given increased ectomycorrhizal inoculum of most fungal species will likely persist at least as long as Pinaceae (*Pinus* or *Pseudotsuga*) are present. Even following removal of these trees, spores of *Rhizopogon* and some other early-successional fungi can persist for at least several and potentially many years (Bruns *et al*. 2009; Nguyen *et al*. 2012). At present there are no effective management options to remove invasive fungi once present.

From an applied perspective, removal of *Pinus* at the seedling or early sapling stage appears to be effective in preventing many of the negative effects of invasion and should be a management priority. Where *Pinus* trees dominate the site it may not be practical to restore and maintain the native tussock grassland and shrubland present prior to *Pinus* invasion. However, it is worth noting that the site was originally a forested ecosystem prior to human induced fire starting with Māoris after the 13th century and increasing with Europeans in the 19th century. If the native forest could be restored, potentially by under-planting native *Kunzea* or *Nothofagus* trees before complete *Pinus* tree removal, it would likely be resistant to both exotic grass and *Pinus* invasion. Our results suggest that any such efforts should not expect ectomycorrhizal *Pinus* to facilitate ectomycorrhizal infection of native trees, hence inoculation is likely to be necessary (Dickie *et al*. 2012).

Our findings highlight that belowground legacies can be generated across multiple trophic levels, involving plants, fungi, nematodes and bacteria. In addition, these legacies represent potentially profound biogeochemical disruption with consequent effects on the success or performance of co-occurring native and non-native species. Understanding the persistence and importance of belowground legacies of biological invaders is needed to predict the assembly and function of these increasingly widespread novel ecosystems.

## Sources of Funding

This research was supported by Core funding for Crown Research Institutes from the New Zealand Ministry of Business, Innovation and Employment's Science and Innovation Group, with additional support of IAD by the Bio-Protection Research Centre of Lincoln University.

## Contributions by the Authors

I.A.D., M.G.S.J. and D.A.P. conceived the study; all authors contributed to experimental design: I.A.D., M.G.S.J., K.O., G.W.Y. and K.I.B. gathered soil data; K.I.B. and I.A.D. designed and executed the greenhouse component; D.A.P. and C.W.M. gathered most vegetation data; I.A.D. analysed the data and wrote the paper, with contributions from M.G.S.J., K.O. and D.A.P.; all authors except G.W.Y. helped to revise and improve the text.

## Conflicts of Interest Statement

None declared.

## Supporting Information

The following Supporting Information is available in the online version of this article–

**Table S1.** Plant community cover (summed across height tiers) in each of the four treatments: Seedling-Removal, Sapling-Removal, No-Removal and Tree-Removal of *P. contorta*. A unique six-letter species code (used in Fig. 3), with species, family, growth form and native status in the alphabetical order by species code (National Vegetation Survey: nvs.landcareresearch.co.nz).

Additional Information
