# Supplementary material for: Belowground legacies of Pinus contorta invasion and removal result in multiple mechanisms of invasional meltdown
Source: AoB Plants. 2013 Dec 19;6:plu056. doi: 10.1093/aobpla/plu056 (PMC4240229; doi:10.1093/aobpla/plu056)
Supplement: Additional Information [file supp_6_plu056_index.html]

Belowground legacies of Pinus contorta invasion and removal result in multiple mechanisms of invasional meltdown — Belowground legacies of Pinus contorta invasion and removal result in multiple mechanisms of invasional meltdown — Additional Information 

# Belowground legacies of *Pinus contorta* invasion and removal result in multiple mechanisms of invasional meltdown

## Additional Information

Additional Information

**Files in this Data Supplement:**

- Supporting Information - doc file
