# Supplementary material for: Belowground legacies of Pinus contorta invasion and removal result in multiple mechanisms of invasional meltdown
Source: AoB Plants. 2013 Dec 19;6:plu056. doi: 10.1093/aobpla/plu056 (PMC4240229; doi:10.1093/aobpla/plu056)
Supplement: Additional Information [file supp_plu056_plu056supp.doc]

| **Table S1.** Plant community cover (summed across height tears) by treatment, giving species code (used in Figure 3), species, family, growth form, and native status in alphabetical order by species code. | | | | | | | | |
| --- | --- | --- | --- | --- | --- | --- | --- | --- |
|  |  |  |  |  | Percent cover (sum across tiers) | | | |
| Species code | Species | Family | Functional group | Native status | Seedling-Removal | Sapling-Removal | No-Removal | Tree-Removal |
| ACACAE | *Acaena caesiiglauca* | Rosaceae | Forb | Endemic | 0.00 | 0.36 | 0.25 | 0.00 |
| ACANOV | *Acaena novae-zelandiae* | Rosaceae | Forb | Non-endemic | 0.00 | 0.07 | 1.00 | 0.00 |
| ACIAUR | *Aciphylla aurea* | Apiaceae | Forb | Endemic | 0.58 | 0.93 | 0.58 | 0.90 |
| ACTNOV | *Actinotus novae-zelandiae* | Apiaceae | SubShrub | Endemic | 0.00 | 0.07 | 0.00 | 0.00 |
| AGRCAP | *Agrostis capillaris* | Poaceae | Graminoid | Exotic | 0.50 | 2.36 | 0.75 | 21.60 |
| ALNGLU | *Alnus glutinosa* | Betulaceae | Tree | Exotic | 0.00 | 0.07 | 0.42 | 0.20 |
| ANABEL | *Anaphalioides bellidioides* | Asteraceae | SubShrub | Endemic | 0.00 | 0.00 | 0.08 | 0.00 |
| ANIARO | *Anisotome aromatica* | Apiaceae | Forb | Endemic | 0.50 | 0.57 | 1.17 | 0.50 |
| ANIHAA | *Anisotome haastii* | Apiaceae | Forb | Endemic | 0.00 | 0.00 | 0.08 | 0.00 |
| ANTODO | *Anthoxanthum odoratum* | Poaceae | Graminoid | Exotic | 1.08 | 0.86 | 1.33 | 11.70 |
| APOBIF | *Aporostylis bifolia* | Orchidaceae | Forb | Endemic | 0.00 | 0.00 | 0.08 | 0.00 |
| ARIFRU | *Aristotelia fruticosa* | Elaeocarpaceae | Shrub | Endemic | 0.00 | 0.14 | 0.00 | 0.00 |
| BETPEN | *Betula pendula* | Betulaceae | Tree | Exotic | 0.00 | 0.07 | 0.08 | 0.20 |
| BLEPEN | *Blechnum penna-marina* | Blechnaceae | Fern | Non-endemic | 0.00 | 0.14 | 0.67 | 0.00 |
| BRABEL | *Brachyglottis bellidioides* | Asteraceae | Forb | Endemic | 0.50 | 0.50 | 0.42 | 0.30 |
| BRARAD | *Brachyscome radicata* | Asteraceae | Forb | Endemic | 0.00 | 0.14 | 0.00 | 0.00 |
| BRASIN | *Brachyscome sinclairii* | Asteraceae | Forb | Endemic | 0.50 | 0.50 | 0.08 | 0.00 |
| CELGRA | *Celmisia gracilenta* | Asteraceae | Forb | Endemic | 0.50 | 0.50 | 0.08 | 0.50 |
| CELLYA | *Celmisia lyallii* | Asteraceae | Forb | Endemic | 0.08 | 0.29 | 0.00 | 0.00 |
| CELSPE | *Celmisia spectabilis* | Asteraceae | Forb | Endemic | 0.50 | 0.50 | 0.50 | 0.50 |
| CERFON | *Cerastium fontanum* | Caryophyllaceae | Forb | Exotic | 0.00 | 0.00 | 0.25 | 0.20 |
| CHIMAC | *Chionochloa macra* | Poaceae | Graminoid | Endemic | 3.50 | 1.00 | 0.58 | 1.80 |
| CHIPAL | *Chionochloa pallens* | Poaceae | Graminoid | Endemic | 0.00 | 2.43 | 1.25 | 0.20 |
| COPDUM | *Coprosma* species "p" dumosa | Rubiaceae | Shrub | Endemic | 0.00 | 0.21 | 0.00 | 0.00 |
| COPPET | *Coprosma petriei* | Rubiaceae | Shrub | Endemic | 1.33 | 0.50 | 0.08 | 0.20 |
| COPPRO | *Coprosma propinqua* | Rubiaceae | Tree | Endemic | 0.00 | 0.00 | 0.08 | 0.00 |
| COPROS | *Coprosma* species | Rubiaceae | Mixed | Multiple | 0.00 | 0.00 | 0.17 | 0.00 |
| CYTSCO | *Cytisus scoparius* | Fabaceae | Shrub | Exotic | 0.25 | 0.07 | 0.00 | 0.00 |
| DEYAVE | *Deyeuxia avenoides* | Poaceae | Graminoid | Endemic | 0.08 | 0.36 | 0.00 | 0.30 |
| DEYEUX | *Deyeuxia* species | Poaceae | Graminoid | Multiple | 0.08 | 0.00 | 0.00 | 0.00 |
| DISTOU | *Discaria toumatou* | Rhamnaceae | Tree | Endemic | 0.92 | 0.43 | 0.67 | 0.60 |
| DRALON | *Dracophyllum longifolium* | Epacridaceae | Tree | Endemic | 0.00 | 0.00 | 4.67 | 0.00 |
| DRAPOL | *Dracophyllum politum* | Epacridaceae | Shrub | Endemic | 0.00 | 0.00 | 0.17 | 0.00 |
| DRAPRO | *Dracophyllum pronum* | Epacridaceae | Shrub | Endemic | 2.42 | 4.64 | 1.08 | 0.50 |
| DRAUNI | *Dracophyllum uniflorum* | Epacridaceae | Shrub | Endemic | 33.75 | 46.07 | 6.33 | 8.30 |
| ELYREC | *Elymus rectisetus* | Poaceae | Graminoid | Exotic | 0.17 | 0.29 | 0.00 | 0.20 |
| ELYSOL | *Elymus solandri* | Poaceae | Graminoid | Endemic | 0.25 | 0.57 | 0.33 | 0.80 |
| EPIALS | *Epilobium alsinoides* | Onagraceae | Forb | Endemic | 0.00 | 0.14 | 0.00 | 0.00 |
| EPIMIC | *Epilobium microphyllum* | Onagraceae | Forb | Endemic | 0.17 | 0.07 | 0.08 | 0.10 |
| EUPREV | *Euphrasia revoluta* | Scrophulariaceae | Forb | Endemic | 0.00 | 0.07 | 0.00 | 0.00 |
| FESNOV | *Festuca novae-zelandiae* | Poaceae | Graminoid | Endemic | 0.83 | 0.86 | 0.58 | 1.40 |
| GAUANT | *Gaultheria antipoda* | Ericaceae | Shrub | Endemic | 0.00 | 0.00 | 0.33 | 0.00 |
| GAUCRA | *Gaultheria crassa* | Ericaceae | Shrub | Endemic | 0.08 | 0.07 | 0.33 | 0.00 |
| GAUDEP | *Gaultheria depressa* | Ericaceae | Shrub | Non-endemic | 0.50 | 1.57 | 0.58 | 0.50 |
| GENCOR | *Gentianella corymbifera* | Gentianaceae | Forb | Endemic | 0.42 | 0.50 | 0.25 | 0.40 |
| GENTIA | *Gentiana* species | Gentianaceae | Forb | Exotic | 0.08 | 0.00 | 0.00 | 0.00 |
| GERSES | *Geranium sessiliflorum* | Geraniaceae | Forb | Endemic | 0.17 | 0.00 | 0.00 | 0.00 |
| GONAGG | *Gonocarpus aggregatus* | Haloragaceae | SubShrub | Endemic | 0.00 | 0.29 | 0.08 | 0.00 |
| GRASS | *Grass species* | Poaceae | Graminoid | Unknown | 0.00 | 0.07 | 0.00 | 0.00 |
| HEBBRA | *Hebe brachysiphon* | Scrophulariaceae | Shrub | Endemic | 1.42 | 1.43 | 1.17 | 0.70 |
| HEBCIL | *Hebe ciliolata* | Scrophulariaceae | Shrub | Endemic | 0.08 | 0.00 | 0.00 | 0.00 |
| HEBODO | *Hebe odora* | Scrophulariaceae | Shrub | Endemic | 0.08 | 0.21 | 0.00 | 0.50 |
| HEBPIN | *Hebe pinguifolia* | Scrophulariaceae | Shrub | Endemic | 0.50 | 0.57 | 0.75 | 0.50 |
| HIECAE | *Hieracium caespitosum* | Asteraceae | Forb | Exotic | 0.50 | 0.71 | 0.08 | 0.60 |
| HIELEP | *Hieracium lepidulum* | Asteraceae | Forb | Exotic | 0.50 | 0.86 | 1.25 | 1.30 |
| HIEPIL | *Hieracium pilosella* | Asteraceae | Forb | Exotic | 8.58 | 4.36 | 1.75 | 19.40 |
| HIEPRA | *Hieracium praealtum* | Asteraceae | Forb | Exotic | 0.92 | 2.79 | 4.92 | 3.30 |
| HOLLAN | *Holcus lanatus* | Poaceae | Graminoid | Exotic | 0.00 | 0.14 | 0.00 | 0.00 |
| HYPRAD | *Hypochaeris radicata* | Asteraceae | Forb | Exotic | 0.50 | 0.50 | 0.08 | 0.50 |
| KELDIE | *Kelleria dieffenbachii* | Thymelaeaceae | SubShrub | Endemic | 0.50 | 0.43 | 0.17 | 0.10 |
| LAGPET | *Lagenifera petiolata* | Asteraceae | Forb | Endemic | 0.17 | 0.29 | 0.00 | 0.00 |
| LAGSTR | *Lagenifera strangulata* | Asteraceae | Forb | Endemic | 0.00 | 0.00 | 0.08 | 0.00 |
| LEPSCO | *Leptospermum scoparium* | Myrtaceae | Tree | Non-endemic | 0.75 | 1.57 | 1.33 | 0.10 |
| LEUCOL | *Leucopogon colensoi* | Epacridaceae | Shrub | Endemic | 0.50 | 0.50 | 0.50 | 0.40 |
| LEUFRA | *Leucopogon fraseri* | Epacridaceae | Shrub | Non-endemic | 0.50 | 0.50 | 0.17 | 0.40 |
| LINCAT | *Linum catharticum* | Linaceae | Forb | Exotic | 0.00 | 0.00 | 0.08 | 0.00 |
| LOTPED | *Lotus pedunculatus* | Fabaceae | Forb | Exotic | 0.08 | 0.21 | 0.00 | 0.00 |
| LUZRUF | *Luzula rufa* | Juncaceae | Graminoid | Endemic | 0.42 | 0.50 | 0.42 | 0.60 |
| LYCFAS | *Lycopodium fastigiatum* | Lycopodiaceae | Fern | Non-endemic | 0.92 | 0.50 | 0.33 | 0.20 |
| LYCSCA | *Lycopodium scariosum* | Lycopodiaceae | Fern | Indigenous | 0.00 | 0.07 | 0.17 | 0.00 |
| MELALP | *Melicytus alpinus* | Violaceae | Shrub | Endemic | 0.00 | 0.07 | 0.00 | 0.00 |
| MICSCA | *Microseris scapigera* | Asteraceae | Forb | Non-endemic | 0.00 | 0.21 | 0.00 | 0.00 |
| MICUNI | *Microtis unifolia* | Orchidaceae | Forb | Endemic | 0.42 | 0.36 | 0.00 | 0.10 |
| MYCMUR | *Mycelis muralis* | Asteraceae | Forb | Exotic | 0.00 | 0.00 | 0.08 | 0.00 |
| NOTCLI | *Fuscospora cliffortioides (=Nothofagus solandri var. cliffortioides)* | Nothofagaceae | Tree | Endemic | 0.00 | 0.14 | 0.00 | 0.00 |
| ORERAM | *Oreomyrrhis ramosa* | Apiaceae | Forb | Non-endemic | 0.00 | 0.07 | 0.00 | 0.00 |
| ORERIG | *Oreomyrrhis rigida* | Apiaceae | Forb | Non-endemic | 0.17 | 0.07 | 0.33 | 0.00 |
| OURCAE | *Ourisia caespitosa* | Scrophulariaceae | Forb | Endemic | 0.17 | 0.14 | 0.00 | 0.00 |
| OZOLEP | *Ozothamnus leptophyllus* | Asteraceae | Shrub | Endemic | 6.33 | 3.86 | 0.17 | 0.30 |
| PIMORE | *Pimelea oreophila* | Thymelaeaceae | SubShrub | Endemic | 0.00 | 0.07 | 0.00 | 0.00 |
| PIMPRO | *Pimelea prostrata* | Thymelaeaceae | SubShrub | Endemic | 0.50 | 0.50 | 0.42 | 0.50 |
| PINCON | *Pinus contorta* | Pinaceae | Tree | Exotic | 0.92 | 20.21 | 147.58 | 2.30 |
| PINNIG | *Pinus nigra* | Pinaceae | Tree | Exotic | 0.00 | 0.00 | 0.00 | 0.70 |
| POACIT | *Poa cita* | Poaceae | Graminoid | Endemic | 0.00 | 0.00 | 0.17 | 0.00 |
| POACOL | *Poa colensoi* | Poaceae | Graminoid | Endemic | 2.58 | 1.21 | 0.33 | 0.50 |
| PODNIV | *Podocarpus nivalis* | Podocarpaceae | Shrub | Endemic | 0.00 | 0.14 | 0.00 | 0.00 |
| PRACOL | *Prasophyllum colensoi* | Orchidaceae | Forb | Endemic | 0.42 | 0.21 | 0.00 | 0.10 |
| PRUVUL | *Prunella vulgaris* | Lamiaceae | Forb | Exotic | 0.00 | 0.07 | 0.00 | 0.00 |
| PSEMEN | *Pseudotsuga menziesii* | Pinaceae | Tree | Exotic | 0.83 | 1.07 | 1.08 | 0.20 |
| RANMUL | *Ranunculus multiscapus* | Ranunculaceae | Forb | Endemic | 0.08 | 0.00 | 0.00 | 0.10 |
| RAOSUB | *Raoulia subulata* | Asteraceae | Forb | Endemic | 0.50 | 0.07 | 0.00 | 0.10 |
| RAOTEN | *Raoulia tenuicaulis* | Asteraceae | Forb | Endemic | 1.25 | 0.43 | 0.25 | 0.30 |
| RUMACE | *Rumex acetosella* | Polygonaceae | Forb | Exotic | 0.00 | 0.00 | 0.00 | 0.20 |
| RYTGRA | *Rytidosperma gracile* | Poaceae | Graminoid | Non-endemic | 0.08 | 0.29 | 0.08 | 0.00 |
| RYTIDO | *Rytidosperma* species | Poaceae | Graminoid | Multiple | 0.00 | 0.07 | 0.00 | 0.00 |
| RYTSET | *Rytidosperma setifolium* | Poaceae | Graminoid | Endemic | 0.50 | 0.50 | 0.08 | 0.30 |
| SCHPAU | *Schoenus pauciflorus* | Cyperaceae | Graminoid | Endemic | 0.00 | 0.36 | 0.00 | 0.00 |
| TRIREP | *Trifolium repens* | Fabaceae | Forb | Exotic | 0.25 | 0.43 | 0.25 | 0.30 |
| VERTHA | *Verbascum thapsus* | Scrophulariaceae | Forb | Exotic | 0.00 | 0.00 | 0.00 | 0.10 |
| VIOCUN | *Viola cunninghamii* | Violaceae | Forb | Non-endemic | 0.00 | 0.43 | 0.25 | 0.20 |
| WAHALB | *Wahlenbergia albomarginata* | Campanulaceae | Forb | Endemic | 0.25 | 0.50 | 0.33 | 0.20 |
